# Susceptibility of SARS-CoV2 infection in children

**DOI:** 10.1007/s00431-023-05184-w

**Published:** 2023-09-13

**Authors:** Nicola Cotugno, Donato Amodio, Danilo Buonsenso, Paolo Palma

**Affiliations:** 1 Clinical Immunology and Vaccinology Unit, Bambino Gesù Children’s Hospital, IRCCS, 00165 Rome, Italy; 2 Chair of Pediatrics, Department of Systems Medicine, University of Rome “Tor Vergata”, Rome, Italy; 3 Department of Woman and Child Health and Public Health, Fondazione Policlinico Universitario A Gemelli IRCCS, Rome, Italy; 4 Centro di Salute Globale, Università Cattolica del Sacro Cuore, Rome, Italy

**Keywords:** Pediatric COVID-19, Long-COVID, COVID-19 genetic susceptibility, COVID-19 vaccines, MIS-C

## Abstract

Coronavirus disease 2019 in children presents with distinct phenotype in comparison to adults. Overall, the pediatric infection with a generally milder clinical course of the acute infection compared to adults still faces several unknown aspects. Specifically, the presence of a wide range of inflammatory manifestations, including multisystem inflammatory syndrome in children (MIS-C), myocarditis, and long COVID in the period after infection suggests a particular susceptibility of some children upon severe acute respiratory syndrome coronavirus 2 (SARS-CoV-2) infection. Albeit peculiar complications such as long covid are less frequent in children compared to adults, research on the relationship between inflammatory syndromes and SARS-CoV-2 is rapidly evolving.

*    Conclusions*: new studies and findings continue to emerge, providing further insights into the underlying mechanisms and potential therapeutic strategies. In the present work, we revised current knowledge of the main factors accounting for such variability upon SARS-CoV-2 infection over the pediatric age group.

**Table:** 

**What is Known:** *• COVID19 in children overall showed a milder course compared to adults during the acute phase of the infection.* *• Children showed to be susceptible to a wide range of post infectious complications including multisystem inflammatory syndrome in children (MIS-C), myocarditis, neuroinflammation, and long COVID. *	
**What is New:** *• Mechanisms underlying susceptibility to a severe course of the infection were recently shown to pertain to the host. * *• A specific combination of HLA was recently shown to be associated to higher susceptibility to MIS-C in children. *

**What is Known:**

*• COVID19 in children overall showed a milder course compared to adults during the acute phase of the infection.*

*• Children showed to be susceptible to a wide range of post infectious complications including multisystem inflammatory syndrome in children (MIS-C), myocarditis, neuroinflammation, and long COVID.
*

**What is New:**

*• Mechanisms underlying susceptibility to a severe course of the infection were recently shown to pertain to the host.
*

*• A specific combination of HLA was recently shown to be associated to higher susceptibility to MIS-C in children.
*

## Introduction

**Fig. 1:**
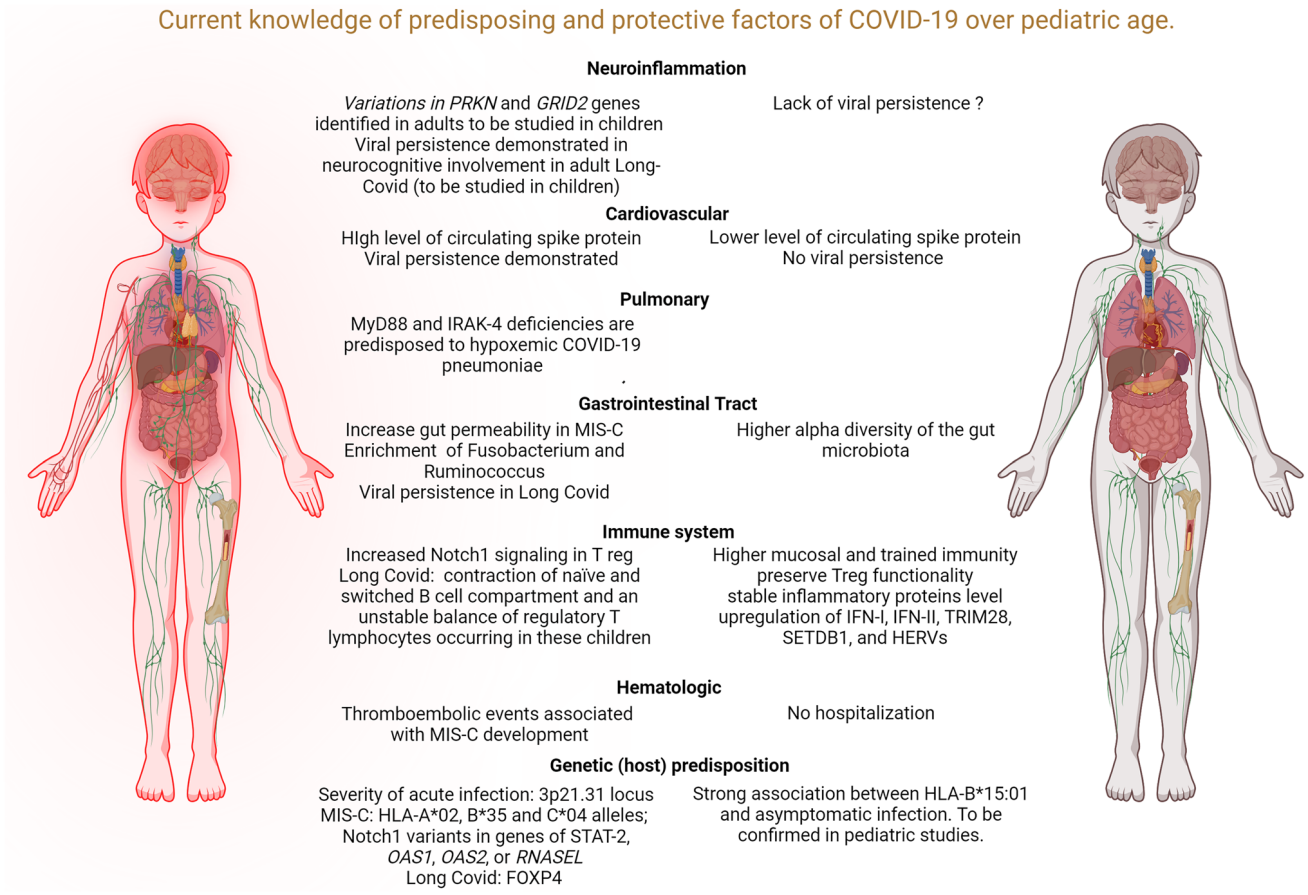
Current knowledge on risk and protective factors for COVID-19 complications in children. The figure outlines the primary pathogenic mechanisms and hypotheses based on organ involvement

Since the emergence of the severe acute respiratory syndrome coronavirus 2 (SARS-CoV-2) in late 2019, our understanding of the virus and its associated clinical manifestations has rapidly evolved [1]. However, the pediatric perspective with a generally milder clinical course of the acute infection compared to adults still faces several unknown aspects. While the primary presentation of COVID-19 is characterized by respiratory symptoms, it has become increasingly evident that SARS-CoV-2 infection can also lead to a wide spectrum of inflammatory syndromes which appear even more problematic in childhood, both in terms of outcome and management, than the infection itself. Understanding factors contributing to these phenomena is crucial to provide new biological insights into disease pathogenesis and to identify mechanistic targets for novel treatments. In this review, we will explore recent insights in relation to the different inflammatory conditions reported in children with/after SARS-CoV2 infection (Fig. 1).

## The acute phase of COVID19 is milder in children compared to adults. Why?

Several uncertainties remain on reasons underlying the distinct and overall milder clinical course experienced by children in the acute phase of COVID19 when compared to adults or elderly [2, 3] . Few hypotheses aiming at deciphering such differences were made and mainly pointing at distinct immune responses upon SARS-CoV2 infection in children. A more vigorous mucosal innate immunity engagement from children was suggested by significantly higher levels of IFN-α2, IFN-γ, and L-1β protein in nasal fluid in children versus adults [4] overall suggesting a protection from the severe disease over the acute phase of the infection. Another hypothesis that emerged over the pandemic was that children may have benefited from a trained immunity due to closer and more frequent contacts with common cold viruses including other coronaviruses and childhood vaccinations [5]. Another possible distinctive feature of the childhood immune system which was hypothesized to underlie the distinctive course of the disease over the acute infection is the naive predominance of the immune response, coupled with a lower cytotoxic T cells in children [2].

Other researchers also aimed at dissecting virological and immunological characteristics in SARS-CoV2 presenting with distinct symptomatology in children. We previously showed how the severity of COVID19 was not related to the extent and persistence of SARS-COV2 specific immunity; however, symptomatic children presented with higher pro-inflammatory cytokines (CXCL10 and CCL19) and longer time to SARS-COV2 clearance [6, 7]. Novel evidence now show that such differences in symptomatology pertain to the host. Indeed, the analysis of 5 distinct HLA loci replicated in two distinct cohort recently identified a strong association between HLA-B*15:01 and asymptomatic infection [8]. Such evidence, investigated in adults, will need to be further confirmed in the pediatric cohort. Another host-related association with symptomatology was also found with microbiota. Although it is unknown whether the higher α diversity found in SARS-CoV2 infected children presenting with no symptoms is either a consequence or a pre-existing feature of this group of children [9], this distinct profile will need to be further explored on larger pediatric cohorts.

## Multisystem inflammatory syndrome in children (MIS-C)

MIS-C is uncommon but represents the most severe inflammatory syndrome observed in children and adolescents following SARS-CoV-2 infection. It typically manifests as persistent fever, along with gastrointestinal symptoms, rash, conjunctivitis, and cardiac abnormalities. Recent evidence suggests that MIS-C may result from an exaggerated immune response triggered by the virus, leading to systemic inflammation and endothelial dysfunction. Vanderbeck and colleagues highlighted the possible role of Notch signaling pathways which have emerged as important regulators of the immune system being at the crossroads of innate and adaptive immunity [10]. Benamar et al. recently showed that MIS-C patients displayed robust T cell activation in association with increased Notch signaling in Tregs. In particular, while Notch4 was also upregulated on circulating Tregs in children with acute COVID-19 as a function of disease severity, the Tregs in those with MIS-C additionally showed upregulation of Notch1 expression, a pathway previously implicated in Th1-skewed immune dysregulation, autoimmunity, graft-versus-host disease, and solid organ rejection [11]. In addition, gastrointestinal-associated events have also been linked with a risk of developing MIC-C in children with previous SARS-CoV-2 infection. Yonker et al. demonstrated zonulin-dependent loss of GI tight junctions in children with MIS-C. The authors hypothesized that in some patients, the presence of SARS-CoV-2 in the GI tract may lead to local mucosal inflammation, increased zonulin release, and a subsequent increase in gut permeability allowing SARS-CoV-2 antigens, including the superantigen-like motif of the spike protein, to traffic across mucosal barriers and into the bloodstream, driving hyperinflammatory immune activation [12]. As an indirect demonstration that uncontrolled inflammation plays a pivotal role in MIS-C, prompt recognition and treatment with anti-inflammatory agents, immunomodulatory therapies, and supportive care have proven crucial in successfully managing this syndrome [13].

## Thrombotic complications and inflammation

In addition to its respiratory and systemic effects, SARS-CoV-2 has been shown to induce a pro-thrombotic state, leading to an increased risk of venous and arterial thrombotic events even in childhood. The incidence of thrombotic events in hospitalized children was much higher in children with systemic inflammation in comparison with children with mild or asymptomatic SARS-CoV-2 infection [14, 15]. The impact on age of this complication was recently explored and pertains mainly to endothelial damage and changes in clotting function, experienced by adults with COVID-19 compared to children [16]. Furthermore, mounting evidence suggest that inflammation plays a crucial role in the pathogenesis of COVID-19-associated coagulopathy. Indeed, evidence of abnormal coagulation profile has also been found in children following the acute phase of infection [17] and at higher extent in children suffering from post-acute complications. Targeting both inflammation and thrombosis pathways holds promise for preventing and managing these complications [15].

## Neuroinflammatory cases in pediatric COVID-19

COVID-19-related neuroinflammation represents an area of growing concern and interest among healthcare professionals and researchers. Although pathogenic mechanisms of such complications have not been fully explored especially in the pediatric context, and SARS-CoV2 is not a classic neurotropic virus, recent findings show the impact of neurological disorders following COVID19 infection in adults [18, 19]. Although children generally experience milder symptoms when infected with COVID-19 compared to adults, there have been reports of neurological complications, including encephalitis, acute disseminated encephalomyelitis (ADEM), acute transversal myelitis, and Guillain-Barré syndrome (GBS) which have been observed in children with COVID-19 [20]. These conditions involve inflammation of the brain or spinal cord and can lead to a range of neurological symptoms such as headache, altered mental status, seizures, weakness, and difficulty walking [21]. The exact mechanisms behind neuroinflammation in pediatric COVID-19 cases are still being investigated, and it is not yet fully understood why certain children are more susceptible to these complications. Further research is crucial to better comprehend the underlying factors contributing to neuroinflammation in pediatric COVID-19 and develop effective strategies for diagnosis, treatment, and prevention of these conditions.

## Long COVID and chronic inflammatory sequelae

A proportion of individuals recovering from acute COVID-19 continue to experience persistent symptoms, known as long COVID. These symptoms often include fatigue, dyspnea, cognitive impairment, and musculoskeletal pain [22]. Emerging evidence suggests that long COVID may be driven by chronic immune dysregulation and a sustained inflammatory response. Understanding the underlying mechanisms and developing targeted interventions are essential to alleviate the burden of long-term sequelae associated with SARS-CoV-2 infection. Long COVID, also addressed by Töpfner et al. in the present issue, is referred as a complex cohort of new-onset signs and symptoms that begins with the infection, or some weeks after, and persist for at least eight weeks and cannot be explained otherwise. The most common symptoms are extreme fatigue, headache, muscle and joint pain, post-exertional malaise, neurocognitive problems, and have a negative impact on the child’s daily functioning (e.g., Attending school or other extra-scholastic activities, or going out with friends) (https://www.who.int/publications/i/item/WHO-2019-nCoV-Post-COVID-19-condition-CA-Clinical-case-definition-2023-1).

So far, pediatric long COVID has been described globally by either researchers or family associations, although its incidence is still unclear since studies used different definitions of long COVID (e.g., symptoms persistence of four, eight, or twelve weeks), designs (e.g., patients assessed in person or through self-filling of online surveys), or populations (admitted or non-hospitalized children) [22]. In fact, while initial studies suggested that 5–15% of children would develop long COVID, a recent and more precise nationwide study performed in Sweden suggested that 0.2% of children that have a known SARS-CoV-2 infection may develop long COVID [23], a much smaller risk compared with a 10% estimated risk in adults [24]. Several independent cohorts have shown that children older than 10 years of age, hospitalized, or with comorbidities bear a higher risk of developing long COVID after SARS-CoV-2 infection [21].

Nevertheless, as for other clinical problems related with SARS-CoV-2, the risk of developing long COVID in children may have changed during the pandemic. For example, severity of acute infection has changed with the surge of new variants and availability of vaccines [25], and risk of MIS-C seems also reduce since the predominance of Omicron and sub-Omicron variants [26, 27]. Similarly, two pediatric studies also found a significant reduction of the risk of developing Long COVID in children infected with the Omicron variant, compared with all previous ones [28, 29].

The change in the risk of developing long COVID with different VOCs is unknown, and current uncertainties about the etiology of this condition make even more difficult to speculate about these differences. So far, a number of events have been associated with risk of long COVID, including SARS-CoV-2 persistence in different tissues [30], alterations in the gut microbiota, chronic immune activation [31], autonomic dysfunction, and chronic vasculitis with circulating micro clots impairing tissue microcirculation and oxygenation [32], vagus nerve inflammation with consequent dysautonomia. Some of these events have also been documented in the first cohorts of deeply investigated children with persisting symptoms after SARS-CoV-2 infection, such as perfusion defects [33, 34], brain hypometabolism [35], chronic immune activation [31], and SARS-CoV-2 persistence [30]. As the pathogenicity of Omicron variants during acute infections has changed compared with the first VOCs, it is possible that also the ability of this VOC to persist in the body or induce chronic vasculitis has changed. In addition, previous immunity (from previous infection or vaccination, or both) may favor a better clearance of the infection, which in turn lead to less risk of developing post-acute sequelae. In adults, in fact, vaccination has been associated with a lower risk of developing long COVID [36]. In children, few studies are available, showing a possible trend toward a lower risk of developing long COVID in fully vaccinated children (at least two doses) [28, 37]. However, as vaccine coverage in children is still very low, most studies have not been able to assess on large enough cohorts if three doses of vaccination significantly protect against long COVID.

Gastrointestinal inflammation in pediatric COVID-19 has been recognized as an important aspect of the disease. While respiratory symptoms are more commonly associated with COVID-19, gastrointestinal symptoms have also been reported, particularly in children. Studies have shown that a significant number of pediatric COVID-19 cases present with gastrointestinal manifestations such as diarrhea, vomiting, and abdominal pain [38, 39].

A retrospective cohort study of 12,306 pediatric COVID-19 patients in the United States [40]. Gastrointestinal inflammation in pediatric COVID-19 is believed to result from the binding of the SARS-CoV-2 virus to ACE2 receptors present in the cells of the gastrointestinal tract. This binding can trigger an inflammatory response, leading to the release of cytokines and activation of immune cells in the gut. The resulting inflammation can cause damage to the intestinal lining and disrupt normal digestive processes. In some cases, pediatric patients with COVID-19 may develop more severe gastrointestinal complications, such as gastrointestinal bleeding, pancreatitis, or bowel perforation. These complications are relatively rare but require prompt medical attention. It is important to note that gastrointestinal symptoms can occur in the absence of respiratory symptoms, making it crucial for healthcare professionals to consider COVID-19 as a possible diagnosis even in children presenting primarily with gastrointestinal complaints [41]. Of note, gastrointestinal involvement in children with SARS-CoV-2 have also been linked with the development of post-acute sequelae, including MIS-C [12] and long COVID [30], and several studies have documented persistence of SARS-CoV-2 antigens in the gut, even months after initial infection [30].

Further research is needed to understand the precise mechanisms and long-term implications of gastrointestinal inflammation in pediatric COVID-19. By gaining a better understanding of this aspect of the disease, healthcare providers can improve diagnostic accuracy, management strategies, and patient outcomes in affected children.

## Post vaccine inflammatory adverse events

COVID-19 vaccines are generally safe and although the benefits of COVID-19 vaccination largely outweigh the risks, rare serious Adverse Events Following Immunizations (AEFIs) have been reported [42, 43]. Apart from the anaphylactic shock following vaccine administration [44], common local and systemic reactions reported for many vaccines, it must be considered the possibility to develop some rare adverse events associated with COVID-19 vaccines. Myocarditis and pericarditis have been reported with a higher frequency in those receiving mRNA COVID-19 vaccination. These cardiac AEFI have been reported especially in male adolescents and young adults [45–49]. Studies from the United States and Israel have indicated that the worldwide occurrence of myocarditis following COVID-19 mRNA vaccination is relatively low, with estimates ranging from 0.3 to 5.0 cases per 100,000 vaccinated individuals [50–52]. Observational data indicates that there might be a higher risk of myocarditis associated with mRNA-1273 compared to BNT162b2. Furthermore, the risk of myocarditis seems to be elevated after the second dose of the vaccine and when the interval between doses is shorter [53–55]. These data highlight the immune-mediated response occurring in myocarditis following vaccination where the immune system reacts to the viral protein fragments introduced by the vaccine, leading to inflammation in the heart muscle. In line with this hypothesis, Yonker et al. recently reported that individuals who developed c-AEFI uniquely exhibit elevated levels of free spike protein in circulation, which appear to correlate with cardiac troponin T levels and innate immune activation with cytokine release [56].

Although most cases have a favorable outcome with a self-limiting course, recent data demonstrate that relapsing myocarditis episodes are also possible [57]. We recently showed a distinct androgens profile and a well restricted inflammation profile on heart in patients developing c-AEFI following mRNA vaccination.

## Genetic insights of inflammatory diseases associated to SARS-CoV2 in childhood

Genetic variations among individuals may influence their susceptibility to developing severe inflammation in response to SARS-CoV-2 infection. Several studies in adults have identified genetic factors associated with an increased risk of severe COVID-19. Among these, the strongest and most robust finding for severity is represented by the 3p21.31 locus. However, few genetic insights are evident for children. Understanding these genetic predispositions can help identify individuals at higher risk and potentially inform targeted treatment strategies. Lee et al. [58] report that variants in genes encoding the 2′-5′-oligoadenylate synthetase (OAS)–ribonuclease L (RNase L) viral RNA sensing pathway lead to exuberant inflammatory responses in myeloid cells in individuals with MIS-C [58]. Sacco et al. have recently validated the association of MIS-C with the combination of the HLA-A*02, B*35 and C*04 alleles arguing for a genetic basis of susceptibility to MIS-C [59]. However, data from Zambrano et al. provides proof of principle for these recent perspectives proposing that genetic risk factors for MIS-C may be incompletely penetrant [60]. Overall future studies with larger cohorts are needed to determine additional genetic risk factors for inflammatory conditions associated with SARS-CoV2 infection because the risk of MIS-C may vary among different genetic causes of autoinflammation. Similarly, a recent preprint from Lammi et al. identified the first genome-wide significant association for Long COVID at the FOXP4 locus. The authors performed a genome-wide association study for long COVID including up to 6,450 long COVID cases and 1,093,995 population controls from 24 studies across 16 countries [61]. They ​​identified a genome-wide significant association within the FOXP4 locus (chr6:41,515,652G > C, GRCh38, rs9367106, as the lead variant), a gene involved in pulmonary function and immune responses. The C allele at rs9367106 was associated with an increased risk of long COVID (OR = 1.63, 95% confidence interval (CI): 1.40–1.89, risk allele frequency = 4.2%).

## Conclusions

Acute and post-acute Inflammatory syndromes associated with SARS-CoV-2 infection represent a significant clinical challenge. It is worth noting that research on the relationship between inflammatory syndromes and SARS-CoV-2 is rapidly evolving. New studies and findings continue to emerge, providing further insights into the underlying mechanisms and potential therapeutic strategies. Recent insights into the pathogenesis of these syndromes have initiated to shed light on the complex and long-lasting interplay between the virus, the immune system, and systemic inflammation, opening new scenarios for a better characterization not only of SARS-CoV-2 associated conditions, but also other common pediatric pathologies like Kawasaki diseases and post-viral chronic fatigue syndromes. Understanding the underlying mechanisms and identifying therapeutic targets are crucial for developing effective preventive and management strategies. Future research should focus on unraveling the pathogenesis and the long-term consequences of these inflammatory syndromes and optimizing interventions to improve outcomes for affected individuals.

## Data Availability

Not applicable.
